# Why women say no to leadership positions

**DOI:** 10.3389/fped.2022.1027295

**Published:** 2022-09-29

**Authors:** Tessie W. October

**Affiliations:** Children's National Hospital, Washington, DC, United States

**Keywords:** academic success, leadership, pediatric ICU, women physicians, career mobility, gender equity, critical care

I am a pediatric critical care physician who followed the academic roadmap by being a strong clinician, trailblazer in research, skilled educator and advocate for my patients. I am no unicorn. I know dozens of women in pediatric critical care who are just like me or better. They are my peers, my academic crushes and my friends. Several of us have considered leadership positions that would require a seismic shift in our lives due to geographical moves, rebalancing of work demands or ceding of our academic joys. Let me be clear: these women are not afraid of change, embrace hard work and are already seen as superwomen who slay challenges. Yet, many of us will not take the leap because the academic ladder was not built for us.

As I considered a leadership position, I searched for women who were succeeding in that role. I struggled to find many role models who were mothers of young children who had relocated to accept that position. When I chatted with a recruiter, she asked, “What will it take for me to move you?” I responded, “The question you should be asking is what motivates me?” She then asked and I responded “Anything I do has to validate my role as a good mother. Secondly, I am motivated by purpose, not fame, not money, not power. Do not obscure my position, I will be compensated for my labor, but if you do not find out what makes me whole it will be a futile process.”

This is not to say men do not care about purpose and family. This is to say that my job as a mother is woven into the fabric of all I do. A geographic move is a heavy undertaking for women because of our outsized role at home. Women bear the onus of resettling the family, establishing the village that makes work-life integration seamless, and managing the emotional toll of disrupting the children's lives. Is there a strong swim team? How long will it take to travel to my parents in an emergency? Will we find a French-speaking nanny? And as a woman of color an extra layer of questions arise around safety. Can my son walk in this neighborhood wearing a hoodie? If I do not know the answers to these questions and the hundreds of other questions playing out in my head, I cannot decide if this job is the right fit. So, while it is important to meet the high-profile, often male decision-makers on that first visit, it is equally important to address these concerns.

My research program centers around teaching communication skills to clinicians and one pearl I teach is that if you approach a family meeting with an agenda and fail to find out what is most important to the family, you will miss an opportunity to learn who they are, how they make decisions and what they value most. Effective goals of care decision making requires asking value-based questions then making recommendations based on the family's values. Without that information you cannot make complex medical decisions, such as placing a tracheostomy. The same is true for life decisions.

A decision as important as a geographic move for a new academic role is complex. The absence of women in front of you adds an additional layer of complexity. Academia has a long history of excluding women from leadership. While pediatrics is dominated by women, the upper ranks are populated by men. In 2019 a gender disparities report on the pediatric critical care workforce revealed that only 32% of division directors and 25% of Department of Pediatrics Chairs are women (1). Women are often overlooked for leadership positions because society has acculturated us to not be seen as innovators. Women are told we are not ready for leadership, so we wait to be selected, to be deemed worthy, to have the door cracked, while men may get an opportunity to stretch for a position in which there is growth. This disparity results in women being considered for leadership positions after they have arrived, have checked all the boxes and are often overqualified for the position. The threat in this approach is that the excitement for the position can be fleeting, the growth in skills incremental and the staying power compromised. In response to these delays, we are told to “lean in,” negotiate for more money, speak up in meetings, or essentially, behave like men. While these strategies work well for men, they are often detrimental for women's careers who are then labeled as aggressive or ungrateful. The system puts the burden on women to change, when most of the challenges we face are systemic and need to be addressed by the organizations we serve. Women do not need to adapt to the system, the system needs to become inclusive and equitable for women such that women have early opportunities to rise into leadership.

A consequence of women not being in the pipeline for leadership positions is that we are shielded from the realities of leadership. We are unacquainted with the negotiation palate of options; is housing a possibility? Can I work remote? What signal does it send if I leave early on Thursdays to attend soccer matches? Can I be my authentic self and still succeed? Women need to be in the room to gain this knowledge while preparing for leadership positions. The system needs transparency. We deserve to know what is possible.

The good news is that many institutions have begun to incorporate some of these elements into recruitment. I remember as I was listening to a Department Chair list the research staff that would be available to me, I dreamed of personal staff who could comparably set me up for success. I fantasized about a personal assistant who could navigate the school landscape, sign me up for local neighborhood listservs, find vets, doctors, and dentists and automate my life similarly to how I curated it in my current setting. I said this out loud and the Chair responded, we have a work-life office that handles that. While the work-life office would not address all the personal tasks, it was a great start. I have also seen institutions begin to set metrics for diversity of race, ethnicity, and gender of applicant pools for leadership positions. These are positive developments, but more needs to be done. If we want more women in academic leadership, especially women of color, academia must bend—speak our language, recruit women earlier in their career, retain them at their home institution, offer salary transparency and recognize the unique challenges of leading while mothering. We can do the job, make us WANT the job.

## Data availability statement

The original contributions presented in the study are included in the article/supplementary material, further inquiries can be directed to the corresponding author.

## Author contributions

TO confirms sole responsibility for the study conception and design, data collection, analysis and interpretation of results, and manuscript preparation.

## Conflict of interest

The author declares that the research was conducted in the absence of any commercial or financial relationships that could be construed as a potential conflict of interest.

## Publisher's note

All claims expressed in this article are solely those of the authors and do not necessarily represent those of their affiliated organizations, or those of the publisher, the editors and the reviewers. Any product that may be evaluated in this article, or claim that may be made by its manufacturer, is not guaranteed or endorsed by the publisher.
